# Antibacterial, antioxidant, and sun protection potential of selected ethno medicinal plants used for skin infections in Uganda

**DOI:** 10.1186/s41182-021-00342-y

**Published:** 2021-06-15

**Authors:** Jane Namukobe, Peter Sekandi, Robert Byamukama, Moses Murungi, Jennifer Nambooze, Yeremiah Ekyibetenga, Christine Betty Nagawa, Savina Asiimwe

**Affiliations:** 1 Department of Chemistry, College of Natural Sciences, Makerere University, P.O. Box 7062, Kampala, Uganda; 2 Department of Forestry, Biodiversity and Tourism, College of Agriculture and Environmental Sciences, Makerere University, P.O. Box 7062, Kampala, Uganda; 3 Department of Plant Sciences, Microbiology & Biotechnology, Makerere University, P.O Box 7062, Kampala, Uganda

**Keywords:** Antibacterial, Antioxidant, Toxicity, Sun protection, Medicinal plants, Skin infections

## Abstract

**Background:**

Rural populations in Uganda rely heavily on medicinal plants for the treatment of bacterial skin infections. However, the efficacy of these medicinal plants for their pharmacological action is not known. The study aimed at evaluating the antibacterial, antioxidant, and sun protection potential of *Spermacoce princeae*, *Psorospermum febrifugum*, *Plectranthus caespitosus*, and *Erlangea tomentosa* extracts.

**Methods:**

The plant samples were extracted by maceration sequentially using hexane, dichloromethane, ethyl acetate, methanol, and distilled water. Antibacterial activity of each extract was carried out using an agar well diffusion assay against *Pseudomonas aeruginosa*, *Staphylococcus aureus*, *Escherichia coli*, *Klebsiella pneumonie*, *Streptococcus pyogenes*, *and Salmonella typhi*. Acute dermal toxicity of the aqueous extract of *S. princeae* and *P. febrifugum*, and *E. tomentosa* was assessed in young adult healthy Wistar albino rats at a dose of 8000 and 10,000 mg/kg body weight. The antioxidant activity of each extract was carried out using a 1,1-diphenyl-2-picryl-hydrazyl (DPPH) radical scavenging assay. The sun protection factor was determined using Shimadzu UltraViolet-Visible double beam spectrophotometer between 290 and 320 nm.

**Results:**

The plant extracts showed good antibacterial activity against the tested bacterial strains with minimum inhibitory concentration (MIC) ranging between 3.12 and 12.5 mg/ml. There was no significant change in the levels of creatinine, alanine aminotransferase, and aspartate aminotransferase in the rats even at a higher dose of 10,000 mg/kg, which was related to the results of biochemical analysis of the blood samples from the treated and control groups. The aqueous and methanol extracts of *S. princeae* showed potential antioxidant properties, with half maximal inhibitory concentration (IC_50_) values of 59.82 and 61.20 μg/ml respectively. The organic and aqueous extracts of *P. caespitosus* showed high levels of protection against Ultraviolet light with sun protection potential values ranging between 30.67 and 37.84.

**Conclusions:**

The study demonstrated that the selected medicinal plants possessed good antibacterial, antioxidant, and sun protection properties. Therefore, the plants are alternative sources of antibacterial, antioxidant, and sun protection agents in managing bacterial skin infections.

## Introduction

Bacterial skin infections are still a burden because of the emerging multi-drug resistant bacterial strains that mainly belong to the genus *Staphylococcus*, *Pseudomonas*, *Escherichia*, and *Klebsiella* [37, 42]. Various topical and oral synthetic drugs such as erythromycin, doxycycline, fluconazole, and cefixime are available for the treatment of skin infections. However, excessive and misuse use of these drugs over a long time has led to the rising resistance of bacteria [2]. Besides being expensive, synthetic drugs have limitations concerning toxicity and side effects such as skin drying, headache, nausea, and loss of appetite [20, 24]. To overcome these limitations, there is a need for the development of effective, safe, and low-cost antibacterial drugs. Exploration of medicinal plant resources may provide valuable leads that can be further developed into antibacterial drugs.

Skin infections such as acne are caused directly or indirectly by oxidative stress initiated by free radicals which are responsible for the occurrence of irritation during acne infection [41]. Besides acne, long exposure of the skin to toxic chemicals, ultraviolet (UV) light, and skin injuries induces production of free radicals in the body. Accumulation of excess free radicals which results into degenerative diseases has also been linked to premature skin aging as well as skin cancer especially in the neglected minority groups like the albinos [18, 21, 29, 41]. Also, a small fraction of UV light (280-320 nm) that escapes through the ozone layer causes skin burns and skin cancers [12]. Since synthetic antioxidants have shown to cause adverse side effects [41], plants are the substitute source of antioxidants that may prevent the development of skin conditions due to UV exposure. Therefore, assessment of the sun protection potential of plant extracts indicates the efficacy and effectiveness of the plant extract in protecting the skin from UV light [12, 34].

Many rural people in Uganda still rely on plant-derived preparations for primary health care needs because they are readily available and cheap. In this paper, we studied species used locally to treat bacterial skin infections and also prevent skin conditions that develop due to UV exposure. Ethnobotanical surveys were carried out by Namukobe et al. [28] and Asiimwe et al. [3]; these reports were based on to select the plants and parts used in this study. For example, *S. princeae* fresh leaves are squeezed and applied on wounds [27, 28], the leaves of *E. tomentosa* are widely used by traditional herbal medicine practitioners to prepare herbal remedies for management of skin infections [25]. The stem bark of *P. febrifugum* powder is mixed in Vaseline for treating skin rashes [40]. Like other *Plectranthus* species, the leaves of *P. caespitosus* have been used to treat stomach infections and skin allergy [3]. Thus, the plants and the parts used were selected based on recorded ethnobotanical knowledge, evidence for their continued wide usage, plant part used, and local availability. It becomes useful to obtain a scientific basis for the possible use of medicinal plants in the treatment of bacterial infections and those associated with oxidative damage. The aim of the study, therefore, was to evaluate the sun protection potential, antibacterial, and antioxidant activities of these selected medicinal plants.

## Materials and methods

### Sample collection and preparation

The plant samples were collected from different parts of Uganda after identification and authentication by a taxonomist from Makerere University, College of Natural Sciences, Department of Plant Science, Microbiology, and Biotechnology. The leaves of *S. princeace* were collected along the shores of Ndura water stream, 2 km along the Makerere University Biological field station, Fort portal. The leaves of *P. caespitosus* and stem bark of *P. febrifugum* were collected from Mpigi District, Misindye Village, Buwama sub-country. The leaves of *E. tomentosa* were collected from Kabale District around Kabale Municipal Council gardens. Voucher specimens for *S. princeace*, *P. caespitosus*, *P. febrifugum*, and *E. tomentosa* were deposited for future reference in account number 50892 with voucher numbers 2, 3, 4, and 5 respectively. The samples were chopped into small pieces and air-dried at room temperature for 28 days. The dry samples were then ground into fine powder sealed in air-tight polythene bags and stored in a cool dry place.

For antibacterial activity, the powdered samples (1.0 kg each) were extracted sequentially by maceration using hexane (Hex), dichloromethane (DCM), ethyl acetate (EtOAc), methanol (MeOH), and distilled water (aqueous). The extraction was carried out five times using 3 L of solvent at each time of extraction. For antioxidant and sun protection potential of *P. caespitosus*, *P. febrifugum*, and *E. tomentosa*, each sample (200 g) was extracted sequentially using a mixture of DCM and MeOH (1:1 v/v) followed by distilled water. Many researchers have extracted antioxidants from plants mainly using polar [11, 23, 38, 41]. Therefore, the organic extracts of these three plants were combined using DCM/MeOH to extract compounds ranging from non-polar to polar. The extracts were filtered through cotton wool followed by Whatman No. 1 filter paper and concentrated to dryness using a rotary evaporator at 40 °C. The dried extracts were transferred to sample bottles and placed in a desiccator containing anhydrous sodium sulfate to remove any traces of water. The dried extracts were later put in tightly stoppered sample bottles and stored in a refrigerator awaiting further analysis.

### Determination of antibacterial activity by agar well diffusion method

#### Preparation of the plant extracts, bacterial strains, and culture media

The crude extract (1.5 g each) was dissolved in 15 ml of dimethyl sulfoxide (DMSO) (for organic extracts) and distilled water (for aqueous extracts) using a vortex mixer to make a stock solution of 100 mg/ml. The bacterial strains tested included sensitive and resistant strains of *S. aureus*, *E. coli*, *K. pneumonie*, *P. aeruginosa*, *S. pyogenes*, and *S. typhi.* The species were selected because they have been reported to be the most prevalent bacteria that cause skin infections and life-threatening multi-drug resistant organisms in skin infections [42]. *S. typhi* is also known for causing cutaneous skin and sternal wound infections [22, 35]. The organisms were obtained from the Department of Pharmacology College of Veterinary Medicine, Animal Resources, and Biosecurity, Makerere University. The organisms were isolated on a nutrient broth, and diluted with 20 ml of the sterile nutrient growth media. The dilutions formed the bacterial stock solutions that were used in the agar-well diffusion assays. Ciprofloxacin (CIP), a standard antibacterial drug was used for control experiments [7].

#### Modified agar well diffusion assay for antibacterial screening

Muller Hinton agar plates were inoculated with test bacteria strains separately by spreading the cultured organism on the surface of solidified agar to obtain a uniform inoculum. Four wells (6 mm in diameter) were punched in the agar. The plant extract (100 μL of 10 mg/ml each) was dispensed into each well. For the control experiment, ciprofloxacin disks (10 μg) were dispensed into the well. The plates were incubated at 37 °C for 24 h. The experiment was performed in triplicate and the diameter of zones of inhibition was measured using a ruler. The results were recorded in millimeters as the average zone of inhibition. Extracts with a zone of inhibition greater than the diameter of a well were considered active [7, 41].

#### Determination of minimum inhibitory concentrations (MIC) by serial dilution

The lowest concentration of the extracts which inhibited the growth of tested bacteria was measured by the MIC using the broth micro-dilution method. Five test tubes were arranged in a rack and labeled 1 to 5. Each test tube was filled with 1.0 ml of DMSO, 1.0 ml of the plant was having a concentration of 100 mgml^−1^ was added to the first test tube. Serial twofold dilutions were made from the second test tube to the 5th test tube to make a concentration of 50.00, 25.00, 12.50, 6.25, and 3.12 mg/ml. Six wells were punched in the agar and labeled according to the dilution order. The diluted extract (100 μl each) was dispensed into each respective well. All experiments were performed in triplicate. The plates were incubated at 37 °C for 24 h. The activity was determined visually by the presence or absence of colonies. MICs were determined as the lowest concentrations of extracts showing clear wells [5].

#### Determination of maximum bactericidal concentrations (MBC) by serial dilution

In determining the MBCs, five test tubes were arranged in a rack and labeled 1 to 5. Each test tube was filled with 1.0 ml of growth media followed by 1.0 ml of the extract having a concentration of 100 mgml^−1^. Serial twofold dilutions were made from the first test tube to the 5 consecutive test tubes to make a concentration of 50.00, 25.00, 12.50, 6.25, and 3.12 mg/ml. The test bacteria (10 μl) was added to each test tube (1-5) and then incubated at 37 °C for 24 h. The agar plate was divided into five partitions and labeled according to the dilution order. The cultured mixture (10 μL) was smeared on each respective partition. All experiments were performed in triplicate. The plates were again incubated at 37 °C for 24 h. Partitions without growth were observed visually and MBCs were recorded as the lowest concentration of the extract that killed the tested bacteria [5].

#### Acute dermal toxicity of the aqueous plant extracts

Acute dermal toxicity assay was carried out using OECD guideline 402 as described by Banerjee et al. [6]. A total of 14 young adult healthy Wistar albino rats weighing 80 to 120 g were divided into 2 groups (treated group/transdermal patch and control/non-treated group). The treated group consisted of 2 animals and non-treated/control group consisted of 1 animal. One day before the acute dermal toxicity started, the backs of rats were clipped with an electric clipper. Each rat was caged individually and left undisturbed for 24 h. The exposed skin was cleaned with non-irritating distilled water. On the test day, the extracts were dissolved in distilled water and applied evenly to the exposed skin at a dose of 8000 and 10,000 mg/kg body weight and covered with a semi-occlusive dressing. Distilled water (3 ml/kg body weight) was topically applied to the exposed skin of the control rats. The rats were then returned to their cages. The animals were observed twice daily for 14 days for signs of irritation, general behavior, and possible mortality. Body weight measurement, food, and water consumptions were taken daily for 14 days. On the 15th day, all animals in the vehicle control and treated groups were killed, organs were carefully taken out and weighed. Histopathological examination of animals was performed at the termination of the study on day 15. The aqueous extracts were selected because water is the common solvent used by traditional herbalist to prepare herbal drugs. Blood for clinical chemistry was placed in vacuum blood collection tubes devoid of anticoagulant (serum tube) and allowed to clot at room temperature. Blood samples were centrifuged at 3000 rpm for 10 min after collection and then the serum was separated. Serum biochemistry parameters including creatinine (CREJ), alanine aminotransferase (ALT), and aspartate aminotransferase (AST) were analyzed by COBAS 6000 analyzer machine. The serum of the experimental rats was compared with those of control rats.

#### Determination of antioxidant activity by 1,1-diphenyl-2-picryl-hydrazyl (DPPH) assay

The antioxidant activity of the crude extracts was determined according to the method of Himesh et al. [16] with slight modifications. Each crude extract (11 mg) was dissolved in 100 ml of methanol for organic extract and water for the aqueous extract to make 110 μg/ml stock solution. Ascorbic acid was used as a standard and was prepared in the same way as the extracts using distilled water. DPPH solution of concentration 0.5 mM and 0.1 mM was prepared and kept in darkness for 45 min at room temperature. The scavenging activity of *S. princeae* was measured using 0.5 mM whereas 0.1 mM of DPPH was used to measure the scavenging activity of *P. caespitosus*, *P. febrifugum*, and *E. tomentosa*. The sample solution (2 ml) was pipetted and mixed with DPPH (2 ml) solution in a cuvette. The mixture was kept in darkness for 15 min to stabilize. The absorbance of the mixture was measured at 517 nm using Shimadzu UV-VIS double beam spectrophotometer against a blank. The percentage inhibition of radicals was calculated using the following formula;

$$ \%\mathrm{inhibition}=\frac{\mathrm{Control}\ \mathrm{Absorbance}-\mathrm{Sample}\ \mathrm{Absorbance}}{\mathrm{Control}\ \mathrm{Absorbance}} $$

Control Absorbance is the absorbance of DPPH only and Sample Absorbance is the absorbance of sample mixed with DPPH. The stock solutions were serially diluted five times and the antioxidant activity of the diluted solutions was determined. The concentration of the extract (antioxidant) required to decrease the initial DPPH concentration by 50% (IC_50_) was calculated using the Logit regression analysis. A lower IC_50_ value corresponded to a larger scavenging power. All experiments were conducted in triplicate and values expressed as mean ± standard deviation (SD).

#### Sun protection potential

The sun protection factor was determined using a modified method reported by Dutra et al. [12]. Each crude extract (0.1 g) was dissolved in 50 ml of ethanol to make a solution of concentration 2 mg/ml without ultra-sonication. The absorption data of each sample was measured using Shimadzu UV-VIS double beam spectrophotometer against ethanol as a blank. The absorption data were obtained for every 5 nm interval between the range of 290 to 320 nm, and four determinations were made at each point and the sun protection factor was determined using the Mansur equation.

$$ {\mathrm{SPF}}_{\mathrm{spectrophotometric}}=\mathrm{CF}\times {\sum}_{\lambda =290}^{\lambda =320} EE\left(\lambda \right)\times I\left(\lambda \right)\times Abs\left(\ddot{\mathrm{e}}\right) $$

Where CF is the correction factor (=10), EE is the erythemal effect spectrum, I is the solar intensity, and Abs is the absorbance.

### Data analysis

The data were analyzed using simple descriptive statistics in Microsoft Excel. The results were expressed as mean ± standard deviation (SD).

## Results

### Antibacterial activity of the plant extracts

In this study, extracts of the four plants were analyzed for antibacterial activity. The zones of inhibition diameters of all plant extracts are shown in Table 1. The range of the inhibition zone was between 10 and 20 mm among the plants screened. The aqueous and EtOAc extract of *P. caespitosus* showed the highest zone of inhibition against *P. aeruginosa* at 20.0 and 20.7 mm respectively. Extracts of *S. princeae* showed the least zones of inhibition diameter against the tested bacteria followed by *E. tomentosa* and *P. febrifugum* extracts with an average inhibition zone of 12.82, 13.15, and 14.70 mm respectively. The data indicated that the extracts displayed a variable degree of antibacterial activity on different tested organisms and the tested strains exhibited variable sensitivity against extracts. All hexane extracts of *S. princeae*, *E. tomentosa*, and *P. febrifugum* were not active.

**Table 1:** Zone of inhibition of the different plant extracts against *S. aureus*, *E. coli*, *K. pneumonie*, *P. aeruginosa*, *S. pyogenes*, *and S. typhi*

Extracts		Diameter zone of inhibition ± SD (mm)					
		*S. aureus*	*E. coli*	*K. pneumonie*	*P. aeruginosa*	*S. pyogenes*	*S. typhi*
SP	Hex	NA	NA	NA	NA		
	EtOAc	NA	NA	NA	NA		
	MeOH	12.2 ± 0.2	NA	11.3 ± 0.3	12.0 ± 0.7		
	Aqueous	NA	NA	NA	15.8 ± 0.8		
	CIP	32.0	34.0	34.0	36.0		
ET	Hex	NA	NA	NA			NA
	DCM	NA	17.3 ± 0.6	NA			10.0 ± 0.0
	EtOAc	16.0 ± 1.0	17.3 ± 0.6	11.7 ± 0.6			13.7 ± 0.6
	MeOH	15.0 ± 1.0	10.3 ± 0.6	14.7 ± 0.6			10.0 ± 0.0
	Aqueous	NA	10.0 ± 0.0	10.0 ± 0.0			15.0 ± 0.0
	CIP	25.0	35.0	32.0			22.0
PC	Hex	17.0 ± 1.0	12.6 ± 0.9	16.7 ± 0.5	17.1 ± 0.8		13.6 ± 0.5
	EtOAc	NA	13.0 ± 0.8	NA	20.7 ± 1.6		15.0 ± 0.0
	MeOH	18.5 ± 0.6	13.3 ± 0.5	16.6 ± 0.4	16.9 ± 0.4		NA
	Aqueous	15.0 ± 0.0	NA		20.0 ± 0.8		19.0 ± 0.0
	CIP	25	32		27		28.0
PF	Hex	NA	NA	NA	NA	NA	NA
	EtOAc	19.1 ± 0.1	15.5 ± 0.2	NA	14.1 ± 0.2	18.3 ± 0.1	NA
	MeOH	14.1 ± 0.1	10.7 ± 0.2	NA	NA	15.6 ± 0.2	NA
	Aqueous	NA	NA	12.0 ± 0.0	NA	NA	13.0 ± 0.0
	CIP	41.7	40.3		40.5	41.7	

*NA* not active (< 6 mm), *CIP* ciprofloxacin, *SP Spermacoce princeae*, *PF Psorospermum febrifugum*, *PC Plectranthus caespitosus*, *ET Erlangea tomentosa*. Blanks – not selected

The MICs of the plant extracts were determined using the broth microdilution method. This assay was performed only for those extracts which gave a significant zone of inhibition against the tested bacterial species. Table 2 displays the MICs and MBCs (in brackets) of the active plant extracts against each bacterial species. The inhibitory property of the extracts was observed within a range of concentration 6.25 to 50.00 mgml^−1^. Among the extracts analyzed, the lowest MIC value of 6.25 mgml^−1^ was obtained against *S. aureus*, *K. pneumonie*, *S. typhi*, and *P. aeruginosa* with extracts of *P. febrifugum*, *S. princeae*, and *P. caespitosus.* The highest MIC value of 50.00 mgml^−1^ was obtained mainly against *S. typhi and E. coli* with extracts of *P. febrifugum*, *E. tomentosa*, and *P. caespitosus*. The MBCs of the tested plant extracts were generally higher than the corresponding MICs, and ranged between 12.50 and 50.00 mgml^−1^. The best MBC (12.50 mgml^−1^) was obtained with EtOAc extract of *P. febrifugum* against *S. aureus*. The lower the MIC or MBC value, the better the activity of the extract. From Table 2, extracts displayed good (MIC = 6.25 mgml^−1^), moderate (MIC = 12.50 mgml^−1^) and low (MIC = 50.00 mgml^−1^) activity.

**Table 2:** Minimum inhibitory concentration (MIC) and minimum bactericidal concentration (MBC) of the plant extracts against tested bacterial strains

MIC (mg/ml) and MBC in brackets							
Plant extract		***S. aureus***	***E. coli***	***K. pneumonie***	***P. aeruginosa***	***S. pyogenes***	***S. typhi***
**SP**	MeOH	12.50 (50.0)		6.25 (25.00)	6.25 (25.0)		
	Aqueous	6.25		ND	ND		
**ET**	DCM		12.50				50.00
	EtOAc	12.50	12.50	12.50			50.00
	MeOH	12.50	50.00	12.50			50.00
**PC**	Hex	ND	25.00	ND	ND		12.50
	EtOAc		50.00		ND		25.00
	MeOH	ND	50.00	ND	25.00		
	Aqueous	50.00			6.25		6.25
**PF**	EtOAc	6.25 (12.50)	50.00 (50.00)		25.00 (50.00)	12.50 (50.00)	
	MeOH	25.00	12.50				
	Aqueous			25.00			25.00

*ND* not detected

### Acute dermal toxicity of the aqueous plant extracts

**Fig. 1:**
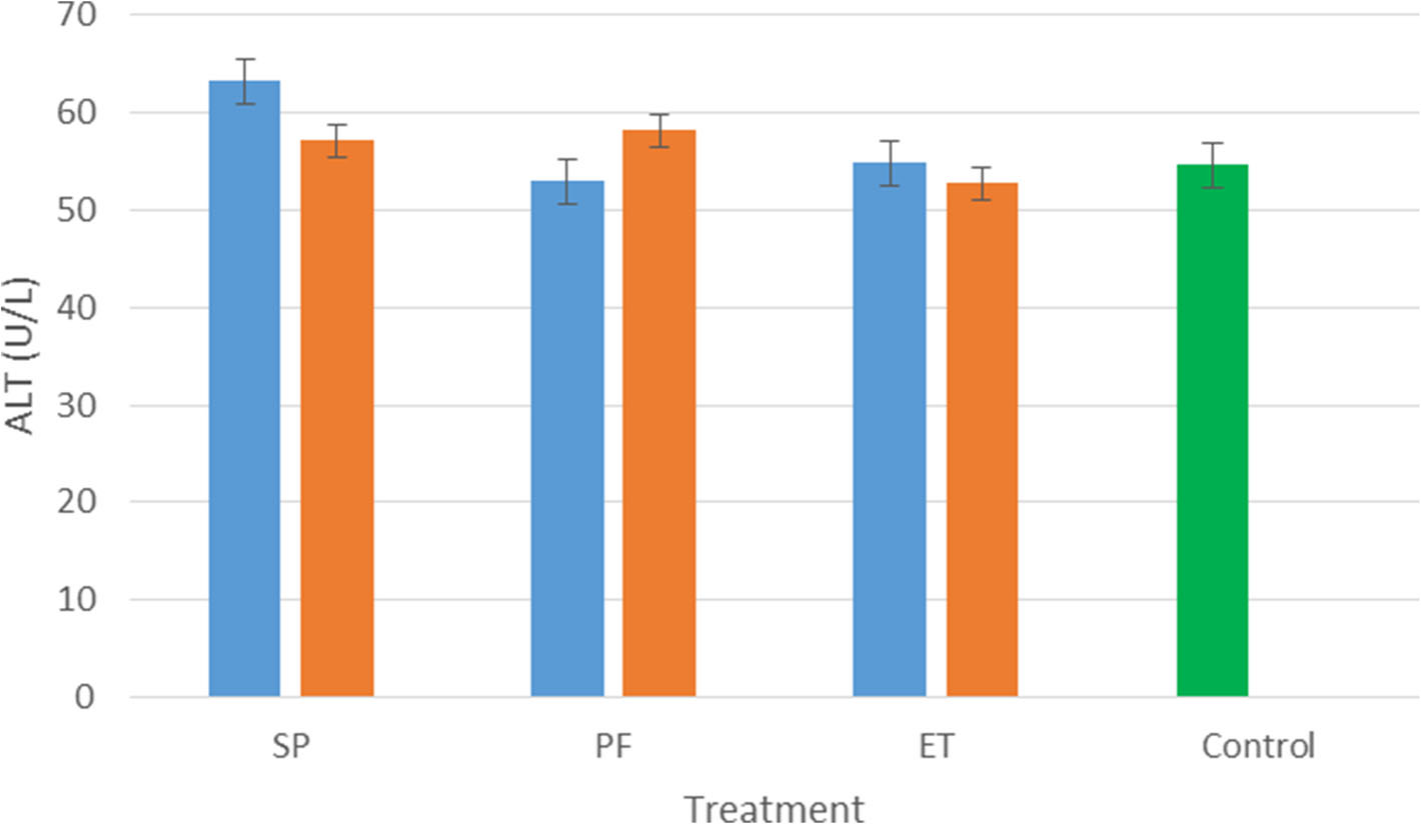
Effect of the aqueous plant extracts on the levels of alanine aminotransferase in the liver

**Fig. 2:**
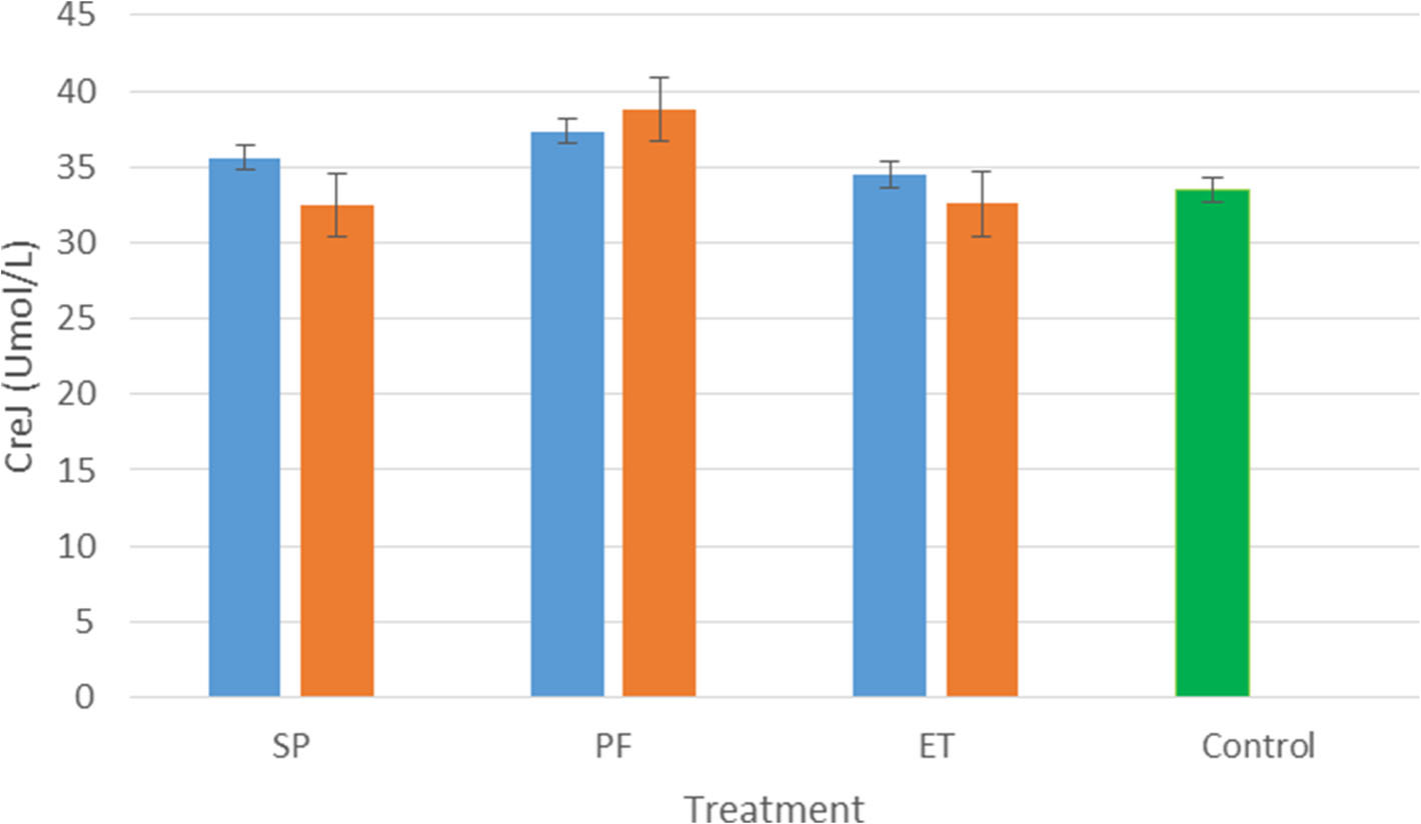
Effect of the aqueous plant extracts on the levels of creatinine in the kidney

**Fig. 3:**
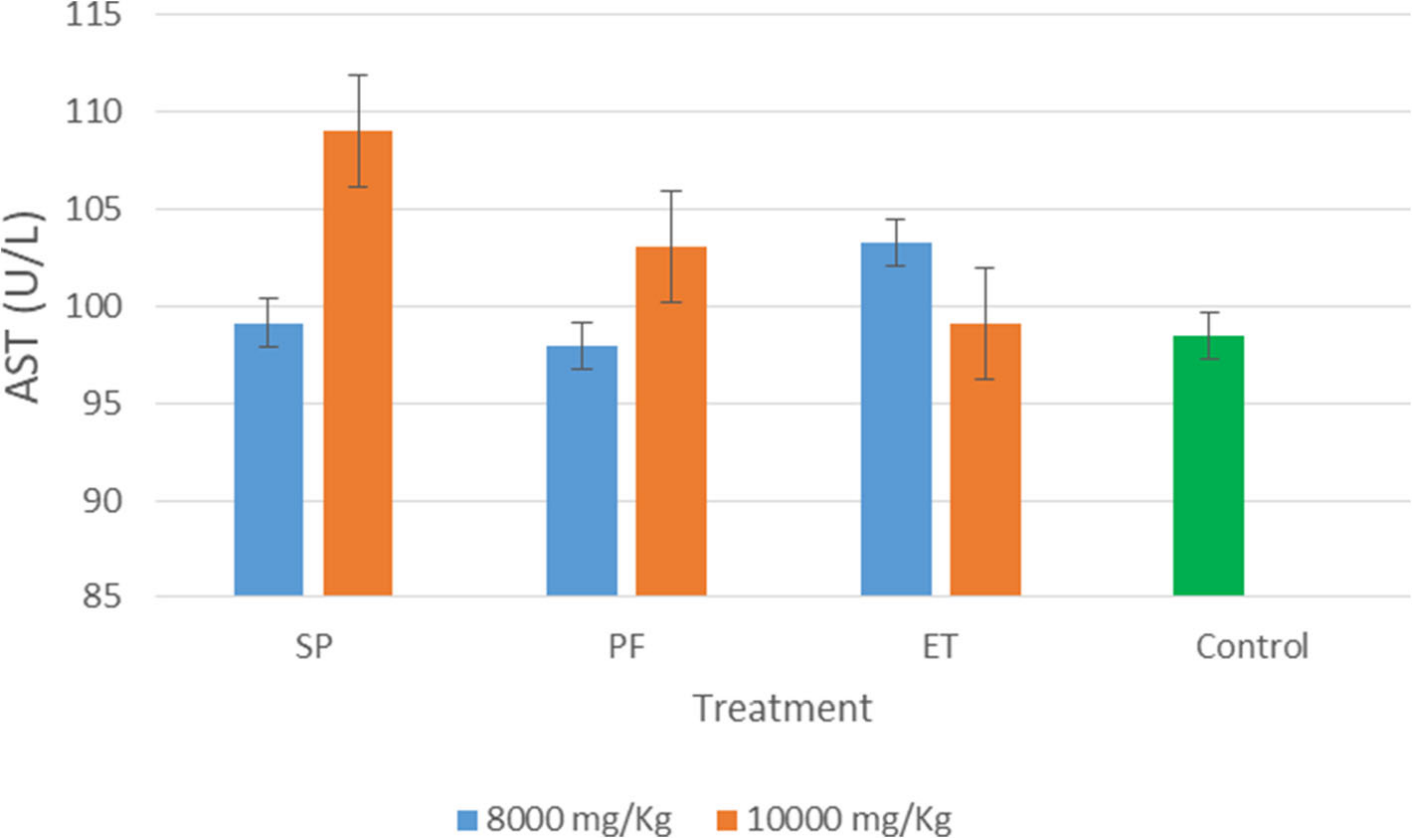
Effect of the aqueous plant extracts on the levels of aspartate aminotransferase in the liver

The results of acute dermal irritation study of the treated and control rats are presented in Figs. 1, 2, and 3. All extracts at both doses have no impact on the levels ALT in the liver except that of *S. princeae* (10,000 mg/kg) which caused a slight increase in the levels of AST in the liver as shown in Fig. 3. Similarly, the extracts had no significant impact on the levels of CREJ in the kidney.

### Antioxidant activity of the plant extracts by DPPH-free radical scavenging activity

Table 3 shows the percentage scavenging activity of the selected medicinal plants and the concentration of each extract needed to decrease the initial DPPH concentration by 50% (IC_50_). All the tested plant extracts showed free radical scavenging activity. The highest radical scavenging activity was shown by *S. princeae* (IC_50_ = 59.82 and 61.26 μg/ml for aqueous and MeOH respectively) and *P. caespitosus* extract (IC_50_, 77.50 μg/ml). Ascorbic acid (IC_50_ = 8.73 and 42.6 μg/ml) was used to determine the effectiveness of the extract in scavenging the free radicals. The closer the IC_50_ value of the plant extract to that of the ascorbic acid, the greater the inhibitory effectiveness [8]. Therefore, *P. caespitosus* and *S. princeae* extracts were more effective in scavenging free radicals than *P. febrifugum* and *E*. *tomentosa.*

**Table 3:** DPPH percentage scavenging activity of the crude extracts of the four plant extracts

DPPH percentage scavenging activity (%), IC_50_ μg/ml in brackets					
	Hex. extract	EtOAc. extract	MeOH extract	Aqueous extract	Ascorbic acid
**SP**	36.03 ± 0.06 (17073.00 )	48.81 ± 0.03 (186.41 )	54.61 ± 0.03 (61.26)	57.01 ± 0.07 (59.82)	77.33 ± 0.05 (8.73)
**ET**	25.04 ± 0.00 (172.70)			36.14 ± 0.00 (316.10)	83.25 ± 0.00 (42.60)
**PC**	46.04 ± 0.00 (77.50)			NT	
**PF**	35.83 ± 0.00 (210.80)			33.95 ± 0.00 (107.40)	

*NT* not tested, *n* = 4

### Sun protection potential (SPP)

Sun protection factor is important to quantify the effectiveness of sunscreen being universally accepted thus the efficacy of a sunscreen is usually expressed by the sun protection potential [19]. The sun protection potential of the plant extracts is summarized in Table 4.

**Table 4:** Sun protection factor of the plant extracts

Extract	ET	PC	SP	PF	4-Aminobenzoic acid
**Aqueous**	3.08 ± 1.36	37.84 ± 0.43	17.05 ± 3.52	7.67 ± 0.69	42.06 ± 6.81
**Organic**	16.46 ± 0.59	30.67 ± 8.96	12.03 ± 3.44	16.67 ± 1.66	

Values = mean ± SD, *n* = 4

All plant extracts showed an ability to protect the skin against ultraviolet (UV) light. The SPP of the standard 4-aminobenzoic acid (42.06 ± 6.81), was used to determine the effectiveness of the extract in protecting the skin against UV light.

## Discussion

The plant extracts examined in the present study display promising antibacterial activity levels against the different bacterial strains and at least each plant had an extract that showed activity against one of the bacterial strains tested. The antibacterial activities of the plant extracts varied according to the solvent used and bacterial strain tested. The methanol extracts were more active compared to other extracts, this could indicate that the active compounds are polar and therefore more soluble in methanol than other solvents [10]. Because the plant gave significant MIC against the tested bacterial species, they can be used for antibacterial herbal formulations. Antibacterial substances are considered as bactericidal agents when the ratio of MBC/MIC is ≤ 4 and bacteriostatic agents when the ratio of MBC/MIC is greater than 4 [1, 41]. Therefore, extracts of *S. princeae* and *P. febrifugum* were bactericidal against the tested bacterial species. The selected plants showed antibacterial activity similar to plants of the same genus in the published literature. For example, *S. verticillata* ethanolic extracts exhibited antibacterial activity against *S. aureus* and *E. coli* at MIC of 100 μg/ml [32], *P. amboinicus* have been reported to exhibit antimicrobial activity against *S. typhi*, *P. aeruginosa*, and *E. coli* at a concentration of 50 mg/ml [36]. Methanolic extract of *P. febrifugum* showed activity against *S. aureus* and *S. epidermidis* with MIC ranging between 6.25 and 50 mg/ml [14]. The bioactivity of the plant extracts could be attributed to the presence of secondary metabolites. For instance, phytochemical screening of *S. princeae* revealed the presence of flavonoids, tannins, saponins, alkaloids, and glycosides [14, 26, 31]. *E*. *tomentosa* has been reported to contain resins, anthraquinones, and coumarins [25]. Also, plants belonging to the genus *Plectranthus* have been reported to contain free aglycone and triterpenes [13]. Flavonoids have been documented to possess a wide range of biological activities such as antioxidant, antibacterial, and sunscreen activities [9] while alkaloids possess bactericidal effect [14]. A study carried out by Nagawa et al. [26] indicated that the stem bark of *P*. *febrifugum* contains volatile compounds such as β-Myrcene, 1,2-Tetradecanediol among others. These essential oils may be responsible for the antibacterial activity of *P*. *febrifugum*.

The liver is a vital organ of the rat which carry out its activities using energy in the form of adenosine triphosphate (ATP). ATP decreases whenever there is any damage in liver. Reduced levels of ATP stimulates the release of enzymes in the tissues; thus, increasing the level of enzymes in the serum whenever the liver is damaged. On the other hand, the low levels of creatinine indicates the inability of kidneys to filter the waste products from the blood and excrete them through the urine [15, 17]. Therefore, the study suggests that at any dermal dose (8000 or 10000 mg/kg), *S. princeae* and *P. febrifugum* are therapeutically safe for liver and kidney, since the experiments showed that the patch-treated groups did not show any biochemical changes up to 14 days of treatment period as compared to the control. On the other hand, the toxic effect of extracts on kidneys was assessed by determining the level of serum creatinine. According to Hossain, the low level of serum creatinine indicates the inability of kidneys to filter the waste products from the blood and excrete them through the urine. Extravenous administration of different doses (up to 10,000 mg/kg) of the extract for 14 days showed no statistically significant difference in the level of serum creatinine. Hence, all the aqueous plant extracts did not show any toxic effect to kidneys. In compulsion to similar studies carried out by other researchers, aqueous extract of *S. princeae* is used by traditional healers to treat bacterial infections and is non-toxic even when administered orally [30]. According to Ntemafack, aqueous extract of *S. princeae* contains some compounds that may either prolong the intestinal transit, excite directly the central nervous system, or possesses nephroprotective and hematoprotective effect when administered orally at middle doses. According to Muhwana et al. [25], the aqueous leaf extract of *E. tomentosa* is safe when orally administered in a single dose (up to 5000 mg/kg) within 24 h. The stem bark of *P*. *febrifugum* is reported non-toxic even at higher dose of 5000 mg/kg body weight [4].

The antioxidant activity is an indicator that the extracts have compounds that could donate hydrogen to the free radical thus serving as radical scavengers [23]. The antioxidant activity of the selected plants was comparable to plants from the same genus. For example, *S. articular* and *S. exilis* have inhibited nitric oxide accumulation in cell cultures at 125 μg/ml [33], methanolic extracts of *P. febrifugum* exhibited antioxidant activity at 0.02 mg/ml [14] whereas *P. amboinicus* has shown promising antioxidant activity at 112.39 μg/ml [39].

Categories of sunscreens are based on protection levels: maximum protection (> 50), high protection (30-50), medium protection (15-30), and low protection (2-15) [34]. From this classification, *P. caespitosus* extracts showed high level of protection against UV light (37.84 ± 0.43, 30.67 ± 8.96). *S. princeae* aqueous, *P. febrifugum* organic, and *E. tomentosa* organic extract showed a medium level of protection with SPP of 17.05 ± 3.52, 16.67 ± 1.66, and 16.46 ± 0.59 respectively. The rest of the extracts exhibited a low level of protection against UV light. The sun protection potential of the selected plants in this study was better than some herbal oils used in cosmetics such as olive (7.549) and coconut (7.119) oil [19]. Extracts from *P. amboinicus* have been also reported to have a low sun protection potential at 12.63 [39]. The obtained antibacterial, antioxidant, and sun protection potential of the plants and other reported bioactivities in literature could explain the use of the plants in the management of skin infections.

## Conclusion

The results of this study indicate that extracts of *S. princeae*, *E. tomentosa*, *P. caespitosus*, and *P. febrifugum* were active against the tested bacterial strains. They are also capable of removing free radicals from the body and can protect the skin from UV light. The results also indicated that the extracts are not toxic (up to 10000 mg/kg) and therefore, the plant can be recommended for application in treating skin infection. The results also showed that polar solvents such as water can extract active compounds with possible free radical scavenging, antibacterial, and sunscreen potential. This information justifies the inclusion of these plants in remedies traditionally used for the management of skin infections and preventing skin conditions associated with exposure of the skin to UV light. The sun protection potential of the plants and antibacterial activity of *S. princeae* and *E. tomentosa* have been reported for the first time. Although the bioassays revealed substantial pharmacological activities, toxicity studies of these crude extracts are being carried out to establish their selectivity. It is also important to isolate and identify the active compounds from the crude extracts.

## Data Availability

Supporting data to this article is publicly available in the Mendeley data repository, Data, V1, doi:10.17632/3jtyz87jc7.1

## Abbreviations

DCM: Dichloromethane
EtOAc: Ethyl acetate
MeOH: Methanol
Hex: Hexane
DMSO: Dimethyl sulfoxide
UV: Ultraviolet
DPPH: 1,1-Diphenyl-2-picryl-hydrazyl
VIS: Visible
MIC: Minimum inhibitory concentration
CIP: Ciprofloxacin
MBC: Maximum bactericidal concentration
IC_50_: Extract concentration required to decrease the DPPH concentration by 50%
SSP: Sun protection potential
SD: Standard deviation
SP: *Spermacoce princeae*
PF: *Psorospermum febrifugum*
ET: *Erlangea tomentosa*
No.: Number
AST: Aspartate aminotransferase
ALT: Alanine aminotransferase
CREJ: Creatinine
